# The Risk of Pelvic Inflammatory Disease in Women Infected With Chlamydia (Chlamydia trachomatis): A Literature Review

**DOI:** 10.7759/cureus.66316

**Published:** 2024-08-06

**Authors:** Neha Maqsood, Jessica Daniel, Sophie Forsyth

**Affiliations:** 1 School of Medicine, University of Bristol, Bristol, GBR; 2 Postgraduate Medical Education, Great Western Hospitals NHS Foundation Trust, Swindon, GBR; 3 Sexual Health and HIV, Great Western Hospitals NHS Foundation Trust, Swindon, GBR

**Keywords:** infection, risk, pelvic inflammatory disease, female, chlamydia trachomatis

## Abstract

A literature review was undertaken to examine the risk of developing pelvic inflammatory disease (PID) in women infected with Chlamydia trachomatis. A search of OVID Medline and Embase databases was conducted for studies published between 1946 and 26 June 2023. This review looked solely at prospective cohort study designs and searched reference lists of studies selected for inclusion. This literature review confirmed that C. trachomatisis a key factor in the development of PID in women through different pathogenic mechanisms. However, to reach more firm conclusions on the subject, further prospective cohort studies with a larger cohort size and a longer follow-up time are needed.

## Introduction and background

Chlamydia (C. trachomatis) is one of the most commonly diagnosed bacterial sexually transmitted infections (STI) worldwide in women [1]. It is particularly prevalent in younger women who are sexually active [2]. Chlamydia has been known to cause severe reproductive health outcomes in women, including pelvic inflammatory disease (PID) [3,4].

PID is a clinical syndrome that affects over one million women globally [5]. It results from the ascending spread of pathogens from the vagina and endocervix to the upper genital tract [6]. PID is often asymptomatic in nature, making the condition difficult to diagnose [7]. It is clinically diagnosed based on symptoms such as lower abdominal pain, purulent vaginal discharge, unscheduled bleeding, and dyspareunia [7]. PID is known to lead to serious obstetric complications, which include ectopic pregnancy, pelvic adhesions, and tubal factor infertility [8,9].

Previous mathematical modelling has shown that 22% of women with an untreated Chlamydia infection progress to developing PID [10]. Several retrospective cohort studies have also found an association between C. trachomatis and PID by investigating the prevalence of chlamydial pathogens and antibodies in women already diagnosed with PID [11,12].

There are limited prospective cohort studies in women who are currently diagnosed with C. trachomatis and followed up until the development of PID. Due to the rising cases of chlamydia globally and the dire sequelae of PID, a literature review was conducted assessing the incidence of PID following C. trachomatis infection [13].

## Review

Methodology

This literature review identified a single study type, prospective cohort studies. The electronic databases used for this search were the Ovid Medline and Embase databases. Papers published between 1946 and 26th June 2023 were searched for by combining search strings to identify as many relevant studies as possible. The keywords included the following: *‘*women’, ‘pelvic inflammatory disease’, ‘chlamydia’, ‘chlamydia trachomatis’, ‘cohort’, and ‘population cohort studies’. To ensure that the search strategy did not miss any studies, relevant keywords from the reference lists of the selected studies were reviewed.

Excluded studies were those whose cohort included pregnant women, women with current illnesses, and women who had abortions, as the latter is a known risk factor for developing pelvic inflammatory disease [14]. Studies were also excluded if they considered other pathogens, such as gonorrhoea (Neisseria gonorrhoea), as the primary infectious agent. Descriptive studies and prospective studies with a control group were also excluded. Finally, editorials, letters, and non-English studies were also excluded.

For the purpose of this review, the definition of pelvic inflammatory disease was a clinical diagnosis of either abdominal or pelvic pain, purulent vaginal discharge, or uterine/cervical tenderness.

Data extraction was carried out solely by the author for studies that met the eligibility criteria - women aged 15 years and older who are found to be infected with C. trachomatis and followed up till they develop pelvic inflammatory disease.

Results

Out of the 778 studies found, seven were relevant to the key question and were selected for the final review. Variables from each study were considered, including the year of publication, cohort size, geographical location, method of diagnosing PID, and incidence of chlamydial infection and PID among women. The data extraction is shown in Tables 1-2.

**Table 1:** Data Extraction From Prospective Studies That Followed Women With Chlamydia Trachomatis to Measure the Development of Pelvic Inflammatory Disease PID (Pelvic Inflammatory Disease); CT (Chlamydia trachomatis); CI (Confidence Intervals)

First Author	Sample Size	Setting	Mean Age of Women (Years)	Mean Follow-up Time
Hoenderboom et al. 2019 [15]	13498	Dutch Women Participating in the Chlamydia Screening Implementation Study (CSI)	31.1	8 Years
Reekie et al. 2017 [16]	315123	Western Australian Women	18	10 Years
Hay et al. 2016 [17]	2529	11 Universities and 9 Further Education Colleges in London	20.8	12 Months
Davies et al. 2013 [18]	307	Sex Workers Recruited in London Between 1985 and 1993	26.2	15.7 Months
Kimani et al. 1996 [19]	302	Treatment Clinic in Nairobi, Kenya for Sex Workers	31	17.6 Months
Hook et al. 1994 [20]	1526	2 Baltimore STD Clinics	Unknown	14 Days
Chaim et al. 1992 [21]	860	12 Rural/Kibbutz Communities in Southern Israel	32.7	5 Years

**Table 2:** Results From Prospective Studies That Followed Women With Chlamydia Trachomatis to Measure the Development of Pelvic Inflammatory Disease PID (Pelvic Inflammatory Disease); CT (Chlamydia trachomatis); CI (Confidence Intervals)

First Author	Number of Women Infected with Chlamydia Trachomatis	Method of Diagnosing PID	Incidence Rate of PID
Hoenderboom et al. 2019 [15]	1682	Self-reported episode of inflammation of the ovaries, uterus, and/or fallopian tubes, diagnosed by a medical professional. Women were asked whether the diagnosis was based on reported symptoms, physical examination, laboratory testing (either blood or vaginal swab examination), or laparoscopy. Women were also asked if they had been admitted to the hospital for the PID episode	2.8%
Reekie et al. 2017 [16]	16778	Linked Hospital Admission or Emergency Department Presentation for PID	28.7%
Hay et al. 2016 [17]	114	Questionnaire asking about the development of PID or related symptoms over the past 12 months. This was backed by medical record search and physical examination by blinded genitourinary physicians	28.0%
Davies et al. 2013 [18]	50	PID was diagnosed at the Sexual Health Clinic using the following clinical criteria: presence of cervical motion, uterine or adnexal tenderness together with more than 5 leucocytes on endocervical gram stain, with or without pyrexia, and with or without lower abdominal pain	16.0%
Kimani et al. 1996 [19]	146	Presence of new pelvic and adnexal tenderness on vaginal examination	15.7%
Hook et al. 1994 [20]	100	Clinical Diagnosis	3.0%
Chaim et al. 1992 [21]	21	Pelvic Examination and Colposcopy	33.3%

The studies focused on women of reproductive age and had significant sample sizes. All studies showed a strong association between C. trachomatis and PID.

Hoenderboom et al. [15] estimated the risk factors of PID in women previously tested for chlamydia in the Chlamydia Screening Implementation Study (CSI) and participating in the Netherlands Chlamydia Cohort Study (NECCST). With a lengthy follow-up time of eight years, Hoenderboom et al. [15] found that women who were chlamydia positive had a higher incidence rate of 4.4 episodes of PID per 1000 person-years (95% CI: 3.3-5.7).

Reekie et al. [16] looked at a significantly large cohort (315,123) of Western Australian women and probabilistically linked chlamydia testing records to hospitalizations and emergency department presentations for PID from 2002 to 2013. They found that the relative risk of PID was 1.77 (95% CI: 1.61-1.94) in women who were chlamydia positive. Reekie et al. [16] also revealed that the incidence of PID was the highest in women co-infected with chlamydia and N. gonorrhoea (incidence rate (IR): 24.4 (95% CI: 21.3-27.5)). This indicates that co-infection with N. gonorrhoea is potentially a stronger risk factor for PID than infection with chlamydia alone.

Hay et al. [17] identified the risk factors for PID in female students across 20 educational settings in London and found that baseline C. trachomatis infection was the strongest risk factor for PID (relative risk (RR): 5.75 (95% CI: 2.63-12.56)). 

Davies et al. [18] and Kimani et al. [19] looked at a cohort of sex workers but in vastly different populations - London and Nairobi - respectively. Both studies had a similar follow-up time and showed a significant association between chlamydia and the risk of developing PID. Davies et al. [18] found that the crude incidence rate of PID in the cohort was 12.0 per 100 person-years (95% CI: 9.0-15.9), whilst Kimani et al. [19] reported a significant relationship between chlamydia infection and PID (adjusted relative risk: 1.8 (95% CI: 1.3-2.4)). They also suggested the risk of PID increases in conjunction with repeated chlamydial infections than an individual chlamydial infection, though they claimed that the power to detect a difference may have been limited.

Other studies, such as Hook et al. [20], which compared a rapid, office-based test with standard cell culture for screening of women for C. trachomatis infections, reported that a delay in acquiring treatment for C. trachomatis increases the risk of developing PID. In the study, 3.2% (95% CI: 0.8-8.5%; three of 93 women) developed PID between the time that they were tested for chlamydia and the time they returned to acquire therapy.

Hook et al. [20] and Hay et al. [17] also showed that women from Black backgrounds were more likely to test positive for chlamydia and possibly be at greater risk of developing PID and other sequelae. Hay et al. [17] also found that women aged less than 20 were more likely to be infected with chlamydia. Chaim et al. [21] listed 'vaginal discharge' and 'adnexal tenderness' - common clinical signs in PID - as obstetric complications in asymptomatic women infected with chlamydia who were followed up for five years.

Discussion

Strengths and Limitations of the Studies

Most studies showed good validity by reducing the chances of bias. In Hay et al. [17], PID was diagnosed via a two-stage process by genitourinary medicine physicians who were blind to the questionnaire, baseline bacteriological tests, and group allocation. Similarly, Hoenderboom et al. [15] adjusted the relation between chlamydia and its sequelae for multiple confounders, including demographic, lifestyle factors, sexual risk behaviour, and marital status of the women. Most studies also reported the attrition value to avoid an overestimation of the PID incidence.

All the studies were conducted in different geographical locations, from Kenya to the Netherlands to Australia to London, thereby increasing the generalisability of the results.

The studies, however, did have some limitations. In Hoenderboom et al. [15], the PID incidence was based on self-reported data possibly creating a diagnostic bias. Another study where there was a risk of this diagnostic bias was by Davies et al. [18] and Kimani et al. [19] study where the population was a cohort of sex workers. Due to the researchers' knowledge of the women’s occupation, there was a risk of leading to any differential diagnosis of lower abdominal pain towards PID. Similarly, Hook et al. [20] also investigated a high-risk demographic in their Baltimore sexual health clinic, where predominately young, Black, women from a low socioeconomic status attended. These studies have ultimately limited the generalisability by reporting a higher-than-normal PID incidence due to a higher-risk cohort.

The selected studies also had varying lengths of follow-up times from 14 days to eight years. Studies where there was a risk of underestimation of PID incidence were in the study by Hook et al. [20], which had a relatively short follow-up time and only had follow-up data for 74% of the chlamydia-positive women. They also did not use the gold standard technique of diagnosing chlamydia (i.e., nucleic acid amplification tests (NAATs)), potentially resulting in an underestimation of the prevalence of chlamydia.

Most studies also lacked primary care data, which could have led to a more accurate value of PID incidence as most mild-to-moderate cases of PID are managed in the primary care setting.

Laparoscopy, which is the gold standard in diagnosing PID, is a very invasive procedure and is not used routinely in clinics. Therefore, most studies diagnosed PID via a clinical history and examination, which could have led to a lower pick-up of PID cases.

Clinicians often make a diagnosis of PID and end up over-treating PID in order to avoid its long-term complications, even though the actual diagnosis is endometriosis, irritable bowel syndrome (IBS), and an ovarian or urinary tract problem. This leads to an overestimation of PID cases in clinical settings.

Within the studies, it would have been beneficial to learn the length of time it took for the women to develop PID after acquiring chlamydia. Understanding the progression rate of PID incidence could help elucidate the pathophysiology of PID and allow screening programmes to optimise their structure and treatment strategy.

Implications for the Future

A major strength of this review is that it has included the most updated research and newer studies. It also attempted to keep the search strategy broad and limit restrictions to review as many publications as possible. The limitations of this paper include the data extraction being carried out by one researcher, which prevented corroboration of the findings. Having a second reviewer could have allowed for further statistical analysis, quality assessment, and critical appraisal of the data. Further, given that it did not investigate grey literature, descriptive, and non-English studies, this review may not be a fully accurate reflection of the complete research landscape, and we may have missed crucial studies relevant to our study question.

The data confirming that higher-risk groups, particularly young and Black women, are more likely to acquire chlamydial infection and its sequelae, should be optimised by screening, high-quality sexual health education programmes, and good access to sexual health services in order to avert long-term sequelae in these patients. Health promotion programmes should be tailored towards these groups who are disproportionately affected by chlamydia and PID. The findings can encourage normalisation of conversations among younger women around protecting against sexually transmitted infections, reduce the stigma and discomfort around testing for these infections, and limit the long-term psychosocial impact of the disease.

NECCST, which is an ongoing cohort study among Dutch women of reproductive age and a follow-up study of the 2008-2011 Chlamydia Screening Implementation (CSI), will follow up with women who have been tested for chlamydia till 2022 [22]. The trial will investigate the sequelae of C. trachomatis infection, and it would be beneficial for future reviews to analyse the updated results, particularly the incidence of PID.

Screening programmes should also consider targeting. Previous research has shown that screening men for sexually transmitted infections can be a cost-effective strategy for reducing PID in women [23]. Further efforts should be made to screen for chlamydia in men and encourage partner notification, which can prevent adverse health outcomes in women.

## Conclusions

To fully comprehend the natural history and sequelae of untreated chlamydial infection, there need to be additional prospective studies with a substantial follow-up time and diverse populations assessing the incidence of PID after chlamydia infection.

The findings from this literature review can potentially help better understand the aetiological role of C. trachomatis and subsequently inform the effectiveness of sexual health screening and control programmes in diverse healthcare settings. C. trachomatis poses a serious health concern, particularly in the developing world, which is already burdened with an unstable healthcare infrastructure. By investing in research that focusses on the aetiology of PID and addressing the gaps in our knowledge, we can develop effective strategies for improving the reproductive health of women globally.
